# Optical Pulling Using Chiral Metalens as a Photonic Probe

**DOI:** 10.3390/nano11123376

**Published:** 2021-12-13

**Authors:** Miao Peng, Hui Luo, Zhaojian Zhang, Tengfang Kuang, Dingbo Chen, Wei Bai, Zhijie Chen, Junbo Yang, Guangzong Xiao

**Affiliations:** 1 College of Advanced Interdisciplinary Studies, National University of Defense Technology, Changsha 410073, China; pengmiao18@nudt.edu.cn (M.P.); lh208@nudt.edu.cn (H.L.); kuangtengfang16@nudt.edu.cn (T.K.); chenzhijie18@nudt.edu.cn (Z.C.); 2 College of Liberal Arts and Sciences, National University of Defense Technology, Changsha 410073, China; zhangzhaojian@nudt.edu.cn (Z.Z.); chendingbo15@nudt.edu.cn (D.C.); 3 CETC Fenghua Information-Equipment Co., Ltd., Taiyuan 030000, China; bw@zdkfh-ie.com

**Keywords:** optical pulling forces, photonic probe, chiral metalens, broadband spectrum, circular dichroism

## Abstract

Optical pulling forces, which can pull objects in the source direction, have emerged as an intensively explored field in recent years. Conventionally, optical pulling forces exerted on objects can be achieved by tailoring the properties of an electromagnetic field, the surrounding environment, or the particles themselves. Recently, the idea of applying conventional lenses or prisms as photonic probes has been proposed to realize an optical pulling force. However, their sizes are far beyond the scope of optical manipulation. Here, we design a chiral metalens as the photonic probe to generate a robust optical pulling force. The induced pulling force exerted on the metalens, characterized by a broadband spectrum over 0.6 μm (from 1.517 to 2.117 μm) bandwidth, reached a maximum value of −83.76 pN/W. Moreover, under the illumination of incident light with different circular polarization states, the longitudinal optical force acting on the metalens showed a circular dichroism response. This means that the longitudinal optical force can be flexibly tuned from a pulling force to a pushing force by controlling the polarization of the incident light. This work could pave the way for a new advanced optical manipulation technique, with potential applications ranging from contactless wafer-scale fabrication to cell assembly and even course control for spacecraft.

## 1. Introduction

Optical manipulations utilizing the mechanical effect of light provide a contactless way to controlling the position of small objects. This phenomenon is attributed to the intensity gradient in the focused field, which can act as an optical trapping potential for confining the particles in three dimensions (3D). Such optical manipulation methods have become essential research tools, with wide-ranging applications in biophysics [1,2], nanotechnology [3,4], classical and quantum physics [5,6] and space science [7,8,9]. Recently, the counterintuitive optical pulling force (OPF) has emerged as an intriguing notion among such optical manipulations due to its remarkable mechanism and potential applications [10,11]. An OPF can pull objects against the light flow by redirecting the incident photons forward, which provides a new manipulation degree of freedom.

OPF acting on particles can be achieved by tailoring the properties of the electromagnetic field, the surrounding environment, or the particles themselves. Various structured optical beams are used to achieve an OPF, including nonparaxial Bessel beams [12,13,14], chiral beams [15,16], and interference of multiple beams [17,18,19]. Alternatively, OPF can also be generated through applying surrounding media with specifically designed properties, such as nonlinear optical liquids [20], topological photonic crystal [21], and waveguide systems [22]. In addition, we can modify the shape and material composition of the photonic probes to obtain the OPF. To date, most studies designing the properties of photonic probes have focused on gain medium [23,24,25], negative refractive index objects [26], chiral particles [27], and core-shell microspheres [28]. The common feature of these methods to realize the OPF is increasing the forward momentum of the scattering field according to the conservation of linear momentum. Alternatively, instead of adding extra forward momentum to the scattered field, one could try from the beginning to input less momentum along the direction of light propagation. A simple, conceptual macroscopic example is the conventional lens or prism, in which the electromagnetic momentum of the incident light can be less than that of the emergent light [29]. However, their bulky sizes are far beyond the scope of optical manipulation.

The advanced fabrication technology of ultrathin and lightweight metasurfaces provides a new solution to the problems mentioned above [30,31]. Metasurfaces are 3D artificial composite nanostructures which exhibit remarkable properties for guiding and controlling the light flow [32,33,34,35]. The change in electromagnetic momentum associated with light scattering also results in previously unexploited optical forces that act on the metasurface itself. A recent work demonstrated that a nanostructured macroscopic object can realize self-stabilizing optical manipulation [9]. The latest work that embedded polarization-sensitive metasurfaces in microscopic metavehicles realized the self-correcting motion [36]. Both works offer an unprecedented opportunity to use the metasurfaces as a unique photonic probe in an optical manipulation system. Among all the applications of metasurfaces, one intriguing development is metalens for wavefront reshaping of light. Due to straightforward fabrication, high transmittance and the possibility of vertical integration, metalens can be potentially replaced or complemented by their conventional counterparts leading to further miniaturization of photonic probes and systems [37,38,39,40]. While the beam-shaping properties of metalenses are well known, the OPF present on metalenses itself is yet to be reported.

In this work, we propose a distinctive paradigm for OPF using chiral metalens as the photonic probe. We designed a Pancharatnam-Berry (P-B) phase-based metalens and simulated the optical force exerted on it using the Finite Element Method (FEM). The numerical aperture (NA) and focal length were taken into account to enhance the OPF efficiently. We obtained the broadband OPF acting on a metalens under the broadband light illumination. Furthermore, due to the chirality of the metalens, it is possible to harness the sign of the longitudinal optical force, i.e., to switch from a pulling to a pushing force, by controlling the circular polarization state of the incident light. Such a feature could be applied to bidirectional particle sorting and transporting in liquids for a greater degree of dynamic control. The potential application domain is spacecraft. The advanced fabrication technology provides opportunities to marry recent developments in ultrathin and lightweight metalenses with grand ambitions for astronautical space travel. Our work is a prospective study on the course control of solar sails. We envision a spacecraft system consisting of a metasurface attached to a satellite to be moved, and whose motion can be controlled by illumination either from stars or high-power, earth-based or satellite-born lasers. Different forces may then be obtained by varying the polarization of the illumination.

## 2. Methods

### 2.1. The Mechanisms of Optical Puling Force Generation

**Figure 1:**
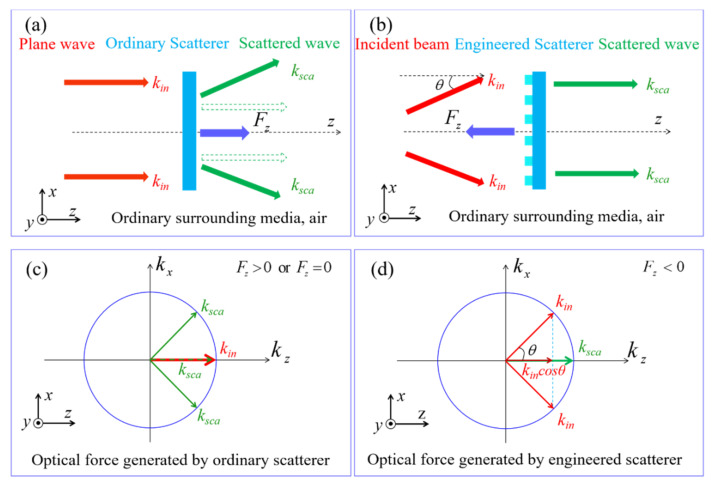
An engineered photonic probe converts the incident waves into waves propagating along the *z*-axis. (**a**) A plane wave with wave vectors (*k_in_*) parallel to the *z*-axis is scattered by an ordinary scatter. (**b**) An incident light with wave vectors (*k_in_*) nonparallel to the *z*-axis is scattered by an engineered scatterer. (**c**) The forward momentum of incident light (red arrow) decreases or remains constant after being scattered by the engineered scatter (green solid and dotted arrow, respectively). Thus, the axis optical force is positive or zero. (**d**) The forward momentum of incident light (red arrow) is enhanced after passing through the engineered scatterer (green arrow), and an OPF is obtained.

**Figure 2:**
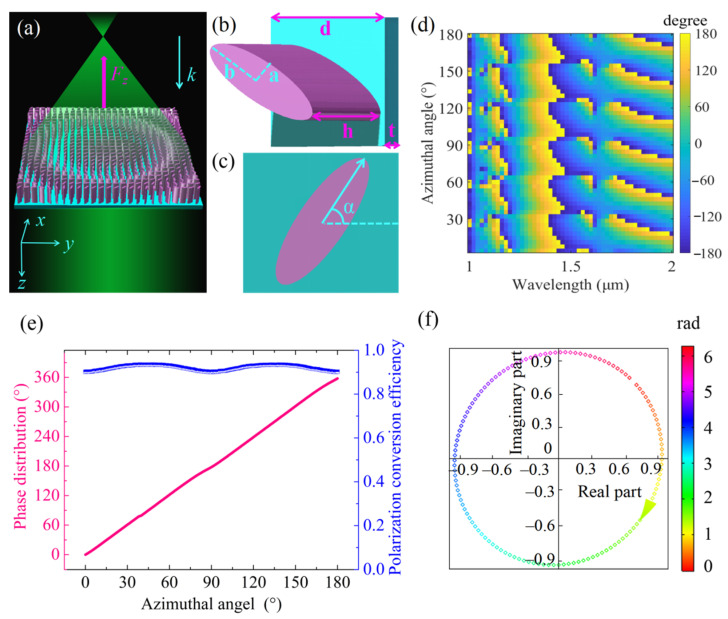
The design process of the metalens photonic probe. (**a**) Schematic of the metalens, where the output beam is steered by metalens to form a collimated beam. (**b**,**c**) Side and top view of a Si nanopillar on the silica substrate, one of the metalens building blocks. We arrange Si nanopillars in a square lattice. For the design wavelength *λ* = 1.817 µm, the unit cell dimension is *d* = 0.8 µm, the substrate thickness is *t* = 0.1 µm, the nanopillar height is *h* = 1 µm, the major axis is *a* = 0.35 µm and the minor axis *b* = 0.1 µm. (**d**) Simulated phase map *φ*(*α*) for the metalens designed at *λ* = 1.817 µm. Each point shows the relative phase difference between a nanopillar and a reference point (the incident light propagates through the vacuum). (**e**) Polarization conversion efficiency (blue squares) and the phase distribution (pink line) of unit cell versus rotating angle *α*. (**f**) Complex transmission coefficients at the designed wavelength.

For plane waves, all the photons travel along the +*z*-direction, which is scattered by the photonic probe. The light momentum changes are not enough to reverse the sign of the scattering forces, as illustrated in Figure 1a,c. Even in an ideal case, where the object is unabsorbed with a 100% transmittance, the axis optical force generated by the incident plane wave only goes to zero, as the green dotted arrow illustrates in Figure 1a,c. Conversely, the desired negative force can be generated if the projection of the incident light momentum along the +*z*-direction is less than that of the output field. By illuminating with wave vectors (*k_in_*) that propagate at angle *θ* along the *z*-axis and use the artificially engineered scatterer (*k_probe_*) to redirect those partial wave vectors (*k_sca_*) along the *z*-axis, one can get the OPF, as illustrated in Figure 1b. In this manner, the light momentum projection in the +*z*-direction increases due to the convex shape of the isofrequency contour, as shown in Figure 1d. According to the law of conservation of linear momentum, the variation of the wave vectors follows *k_in_*cos*θ* = *k_sca_* + *k_probe_*. Therefore, the force exerted on the probes becomes *F_z_* = (*P*/*c*) (cos*θ* − 1), where *P* is the incident optical power illuminating on the probes and *c* is the velocity of light. The force on the probes is negative for *θ* ≠ 0°. From Figure 1b,d, we propose that it is possible to achieve the intrinsic feature of light momentum by using an artificial photonic probe, i.e., a metalens, which can converse the divergent Gaussian beam into the collimated Gaussian beam, as shown in Figure 2a.

### 2.2. The Metalens Photonic Probe Design

The building blocks of a metalens are silicon (Si) nanopillars on the silica substrate, as shown in Figure 2a. When the metalens was illuminated from the Si side with a divergent, left circularly polarized (LCP) Gaussian beam at wavelength *λ* = 1.817 µm, it converted the light into a collimated right circularly polarized (RCP) beam. In order to realize this conversion, every nanopillar had to contribute a phase *φ* at its position (*x*, *y*). The phase *φ* had to follow the principle [41]:

(1) $$\varphi (x,y)=\frac{2\pi }{\lambda }\left(\sqrt{x^{2}+y^{2}+f^{2}}-f\right)$$

where *λ* is the wavelength of the incident light, *f* is the focal length of the metalens. The phase profile *φ* (*x*, *y*) was realized by rotating the azimuthal angel *α* (*x*, *y*) of each unit cell at coordinate (*x*, *y*) using the P-B phase modulation described in Appendix A. The LCP light will therefore be converted into RCP light. Based on the theory mentioned above, we designed a metalens with the following nanopillar structure parameters: major axis *a* = 0.35 µm, minor axis *b* = 0.1 µm, height *h* = 1 µm and unit cell size *d* = 0.8 µm. We assumed the refractive indexes of Si and silica to be 3.48 and 1.45, respectively, at the appropriate wavelength of interest. A detailed description of the design process can be found in Appendix B.

Figure 2d shows the phase *φ* as a function of azimuthal angle *α*, ranging from 1 to 2 μm of the near-infrared band. Full phase coverage (2π) was achieved. The phase modulation and polarization conversion efficiency results are shown in Figure 2e by pink lines and blue squares, respectively. The phase profile linearly covered the whole 2π range with the azimuthal angle rotated from 0 to π. The polarization conversion efficiency of the metalens exceeded 90%. Figure 2f shows the complex transmission coefficients (*T*(*α*)*e^iφ^*^(*α*)^) at the designed wavelength for a range of azimuthal angles required to give 2π phase coverage. Each point in the complex plane represents the phase and amplitude of the transmission of a nanopillar with azimuthal angle *α*. The high transmission, the RCP output field (clockwise arrow) and the phase *φ* coverage approaching 2π are evident for the designed wavelength.

**Figure 3:**
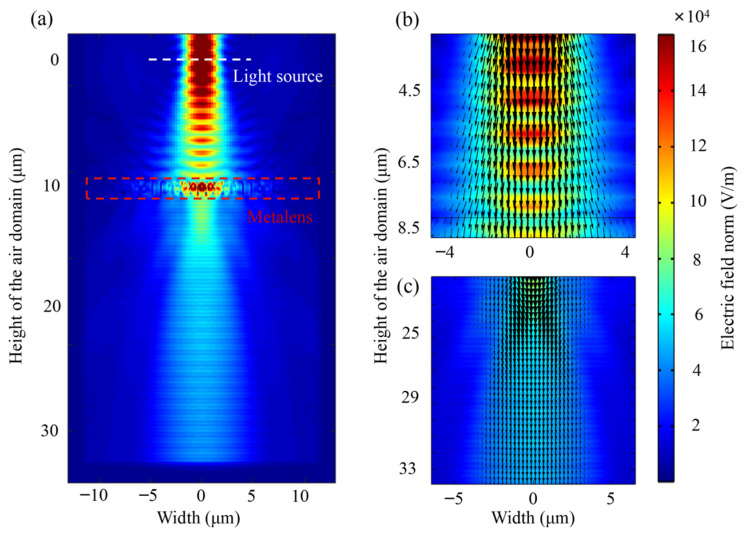
Simulated electric field norm intensity profiles (*y*-*z* plane) of the metalens designed at a wavelength of 1.817 µm focal length of 10 µm. (**a**) Electric field intensity profiles (*y*-*z* plane) of the metalens designed at a wavelength of 1.817 µm and focal length of 10 µm, the position of the light source and the metalens are shown in the white and red dotted lines, respectively. The black arrows indicate wave vectors of (**b**) the incident and (**c**) the output field.

A metalens with a focal length of 10 µm was applied to show the conversion effect from the divergent beam to the collimated beam. Figure 3 shows the electric field intensity distributions of the metalens in the *y*-*z* plane. The divergent LCP Gaussian beam, passing through the metalens, turned into a collimated RCP Gaussian beam. The incident beam propagated along *z*-direction from the Si side with a focal radius of 1.4 µm. The position of the light source and the metalens are indicated by white and red dotted lines, respectively, in Figure 3a. The black arrows shown in Figure 3b,c indicate the direction of the wave vectors for the incident and output field. The collimation efficiency *η* is defined as the ratio of the *z* component of the output electric field to that of the incident electric field. Then, *η* as high as 1.44 may be achieved for the metalens with a focal length of 10 µm.

### 2.3. The Optical Force Calculation

**Figure 4:**
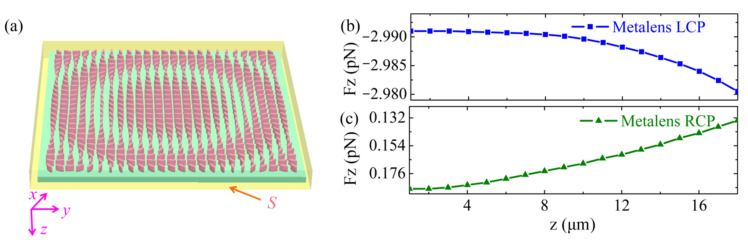
The longitudinal optical force for metalens. (**a**) Schematic of a metalens enclosed by the highlighted surface S. The longitudinal optical force profiles of a metalens illuminated by the divergent (**b**) LCP and (**c**) RCP Gaussian beam in a vacuum, respectively. The curves are interpolated from the calculated data points.

The optical force was evaluated using the Maxwell stress tensor (MST). We considered an electromagnetic wave impinging on a metalens with a unit cell in the *x*-*y* plane, as shown in Figure 4a. The contribution to the total force exerted on the metalens enclosed by the highlighted surface *S* can be calculated as described in [42].

(2) $$\langle F_{i}\rangle =\underset{s}{∯}\langle T_{ij}\rangle n_{j}dS$$

where *n_j_* is the unit vector normal to the surface, *T_ij_* is the time-averaged MST:

(3) 〈Tij〉=12Re[ε0(EiEj*−12δij∑kEkEk*)+μ0(HiHj*−12δij∑kHkHk*)]

where $\underset{k}{\sum }E_{k}E_{k}^{*}$ is expressed as the total electric field. It is noted that *i*, *j* and *k* can take the value of *x*, *y*, *z*, respectively.

We assumed that the lateral (*x-y*) plane of the metalens would be much larger than its transverse (*z*) plane. Therefore, the dominant contribution to the stress tensor was made by the two faces of the integrating surface *S* parallel to the *x-y* plane. Under this condition, we could simplify Equation (2) as

(4) $$\langle F_{i}\rangle =\underset{s}{∯}dxdy\langle T_{ij}\rangle n_{j}$$

## 3. Simulation Results and Discussion

### 3.1. The Optical Force Calculation

To gain more insight into the physical mechanisms for the OPF, we simulated the longitudinal optical force profiles for a metalens illuminated by the divergent LCP and RCP Gaussian beam, respectively, as shown in Figure 4b,c. The incident power *P* was 100 mW at a of wavelength 1.817 µm. The light source was located at the axial position of *z* = 0 µm, with a radius of 2 µm. For both models, the width and height of the structures were 20 and 1.1 µm (for metalens, the thickness of the substrate is 0.1 µm, the height of the nanopillars are 1 µm). The surrounding medium was vacuum. The dynamics of the metalens under divergent LCP Gaussian beam illumination was numerically calculated (see Appendix C).

Intriguingly, because of the chirality of the designed metalens, the longitudinal optical force acting on the metalens exhibited a circular dichroism response under the illumination of incident light with different polarization states. Specifically, an LCP light illumination always exerted an OPF on the metalens, whereas an RCP illumination always pushed the metalens away. The chiral metalens could convert the divergent LCP incident light into collimating RCP light and realize the concentration of optical momentum in the *z*-direction originating an OPF. However, for the case of RCP incident light, the scattered wavefront could not be reshaped, and was absorbed and reflected by the chiral metalens, achieving a pushing force. This meant that we could flexibly tune the longitudinal optical force from a pulling force to a pushing force by controlling the polarization of the incoming light.

### 3.2. Parameter Analysis

**Figure 5:**
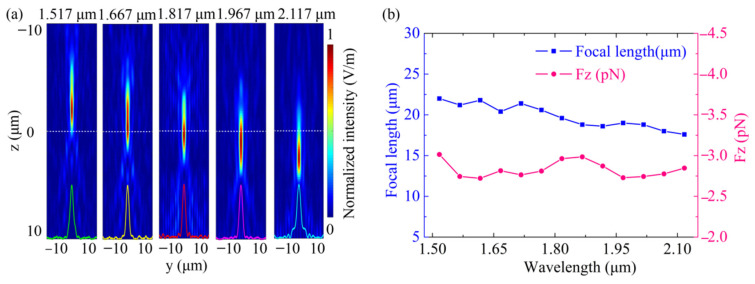
(**a**) Simulated intensity profiles of the focal point in the *y*-*z* plane of the metalens. The wavelengths of the incident field are 1.517, 1.667, 1.817, 1.967 and 2.117 µm, respectively. The inset curves show the normalized intensity profile along the dashed white line. (**b**) The OPF (the pink line) and the focal length (the blue line) as a function of wavelength ranging from 1.517 to 2.117 µm. The metalens has a width of 20 µm, a focal length of 20 µm, NA = 0.4. The position of the metalens is *z* = 20 µm, and the position of the light source is *z* = 0 µm.

For a metalens with 20 µm width, 20 µm focal length and NA = 0.4, the OPF as a function of the wavelength ranging from 1.517 to 2.117 µm was calculated. The position of the metalens was *z* = 20 µm, and the position of the light source was *z* = 0 µm. Figure 5a shows the simulated intensity profiles of the metalens in the *y*-*z* plane, where the incident wavelengths were 1.517, 1.667, 1.617, 1.967 and 2.117 µm, respectively. As we can see, noticeable chromatic aberration can be observed with increasing wavelength, as evidenced by focal length reduction. The normalized intensity profile along the dashed white line exhibited a defocusing property. This was attributed to the dispersion arising from a periodic lattice [39]. Figure 5b shows the dependence of the OPF (pink circles) and the focal length (blue squares) at the incident wavelength for the chromatic case. It should be noted that the OPF was calculated by changing the wavelength and focal length simultaneously. Since the focal length change ∆*f* = 4.4 μm was negligible, the change in OPF was not apparent. However, the OPF curve fluctuated, with a 5.63% total fluctuation value being due to the chromatic aberration.

**Figure 6:**
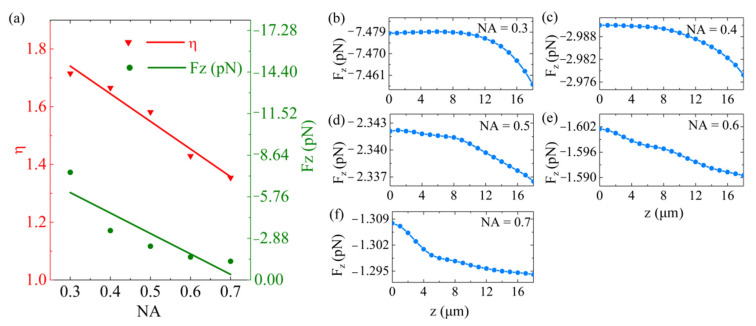
The OPF distributions within a NA range from 0.3 to 0.7. (**a**) Corresponding values for the OPF and the *η*, as a function of the NA. The metalens is located at the axial position *z* = 20 µm. The OPF profiles on the *z*-direction (*x* = *y* = 0) at the NA of (**b**) 0.3, (**c**) 0.4, (**d**) 0.5, (**e**) 0.6 and (**f**) 0.7, respectively.

Figure 6 shows the relation between the OPF and the NAs. We consider that the application scenario of the designed metalens is space, and therefore, did not consider the effect of gravity. Five metalenses were further designed with NA of 0.3, 0.4, 0.5, 0.6 and 0.7, respectively. In this process, the wavelength *λ* and the incident optical power *P*, the focal length *f* and the unit cell parameters of the metalens were assumed to remain constant. Because the NA is proportional to *D*/*f*, we increased D to enhance the NA accordingly. Different NAs have their focal radius *w_0_,* and we varied *w_0_* to match the selected NA. For the five NAs mentioned above, *D* was varied as 15, 20, 25, 35 and 45 μm, and *w_0_* was changed to 3.22, 2.04, 1.82, 1.47 and 1.37 μm, respectively. In Figure 6a, the OPF declined when NA increased. As the power of the incident light was assumed to remain constant, the *D* increased correspondingly with the increase of NA. Hence, the energy distribution per unit area on the metalens decreased. In addition, we found that *η* was reduced with an increase of NA. This meant that the greater the divergence angle of the incident light, the less the outgoing light field was in the *z*-axis, consequently reducing the OPF. For instance, when the metalens was located at an axis position of *z* = 20 µm (*x* = *y* = 0), the OPF was −7.457 and −1.294 pN with respect to 0.3 and 0.7 NA, respectively. Detailed data for each NA are illustrated in Figure 6b–f.

For the metalens with NA = 0.1 and NA = 0.2, the *D* was less than 15 μm and the number of nanopillars on the surface was less than 324. According to Equation (1), the phase profile of the metalens was 0 ~ π. Therefore, the metalens could not modulate the wavefront of the outgoing field, and thus, could not obtain OPF. In addition, the gravity force was greater than the OPF under the circumstance of NA = 0.5, 0.6, 0.7 in a vacuum. For these values, we increased the incident light power to overcome the impact of gravity (See Appendix B).

**Figure 7:**
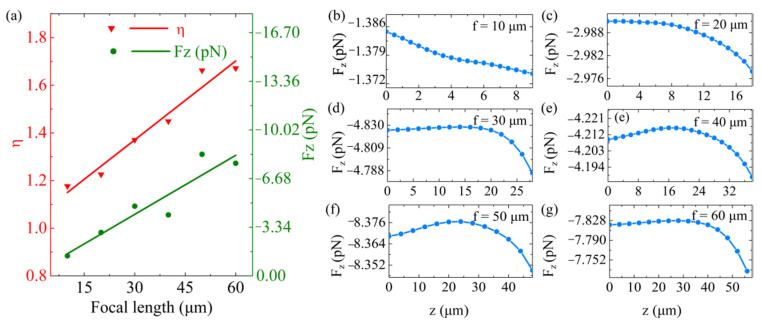
The OPF on the metalenses with different focal lengths *f*. (**a**) The OPF and *η* as a function of *f*. The OPF profiles on the *z*-direction (*x* = *y* = 0) at the *f* of (**b**) 10 µm, (**c**) 20 µm, (**d**) 30 µm, (**e**) 40 µm, (**f**) 50 µm and (**g**) 60 µm, respectively.

The OPF on the metalenses with different focal lengths *f*, including 10, 20, 30, 40, 50 and 60 µm, respectively, was simulated, as shown in Figure 7. We consider that the application scenario of the designed metalens is space, and as such, did not consider the effect of gravity. During the simulation, the wavelength λ and the incident optical power *P* of the incident light, the width *D* and the unit cell parameters of the metalens remained constant. The NA of the metalens and the focal radius *w_0_* of the incident beam were changed to get different *f*. From Figure 7a, we find that the OPF and the *η* curves showed upward trends when *f* increased over a range of 10 to 60 µm. As expected, the *η* was small for the short focal length due to the increasing difficulty of collimating the light to a closer point over the same wavelength [43]. More importantly, the OPF was related to the *η*. As a result, the OPF was enhanced with an increased focal length. We have included further characteristic data for each *f* in Figure 7b–g. In addition, the gravity force was greater than the OPF under the circumstance of *f* = 10 μm in a vaccum. We increased the incident light power to overcome the impact of gravity (See Appendix B).

## 4. Discussion

OPF was achieved by enhancing the forward momentum for the case of previously proposed photonic probes, such as gain media [23,24,25] and plasmonic nanoparticles [44]. In contrast, we reduced the *z* component of incident momentum by applying the metalens as a photonic probe. When the divergent Gaussian beam passed through the metalens, the incident waves that propagated at angle *θ* along the *z*-axis impinged on the metalens. Then, the metalens redirected these waves along the *z*-axis, leading to a dramatically increased backward recoil momentum. As a result of the law of conservation of momentum, the metalens obtained OPF directed toward the light source.

One of the significant advantages of the metalens photonic probe scheme is that it can realize broadband OPF over 0.6 μm bandwidth. OPF was maintained at around −28.3 pN/W at the near-infrared band range, i.e., from 1.517 to 2.117 μm. Conversely, most photonic probes need to be excited at a specific wavelength to obtain OPF, as shown in Table 1. For particles immersed in nonlinear optical liquids, femtosecond laser pulses with a wavelength of 0.42 μm are required, as suggested in [20]. Furthermore, for the gain medium described in [45], a 0.457 μm-wavelength pump laser was required to excite stimulated emission.

Another advantage of the present method is that we can flexibly obtain bidirectional manipulation of the chiral metalens by controlling the handedness of the incident light. Similarly, the chiral slab suggested in [27] can also achieve this manipulation. However, this system needs an extra opaque mirror that reverses the handedness of the incident light. In addition, the chiral slab irradiated by the laser can experience a thermophoretic force due to optically-induced heating. In this work, the absorption in silica and Si is minimized. The flexible tunability of the longitudinal optical force could be applied to the course control of solar sails. Thus, the bowing issue discussed in [8], introduced by flipping the solar sails, could potentially be overcome.

## 5. Conclusions

In summary, we have proposed a novel mechanism to achieve OPF by applying a chiral metalens as an photonic probe. After passing through the metalens, the divergent beam was converted into a collimated beam. The momentum of the outgoing field gathered in the *z*-direction, and was larger than the projection of the incident field momentum in the *z*-direction. As a result of the conservation of momentum, the metalens was subject the recoil momentum and the resultant longitudinal optical force directed toward the light source.

We analyzed the detailed relationships between the OPF and the parameters of the metalens, including wavelength, NA, and focal length. Our research showed that the metalens could achieve OPF across the near-infrared band from 1.517 to 2.117 μm. Meanwhile, decreasing NA or increasing focal length were found to enhance the OPF. The pulling and the pushing force can be switched mutually by controlling the handedness of the incident light.

We envision a number of compelling extensions to this work. Potential applications range from the micro to the macro field. For example, the proposed scheme could be used as a unique photonic probe to realize a polarization-controlled sorting and transport in biological physics. In the macroscopic field, our findings may lead to platforms for pulling macroscopic objects. Incorporating such polarization-controlled manipulation methods in optomechanical systems will allow more manipulation degrees of freedom to probe quantum and classical optomechanics [46] and phase-space dynamics [47]. Furthermore, this work may extend the range of motion of solar sail-based spacecraft. It would be intriguing to combine the polarization-sensitive metalens with a self-stabilizing structure to control the course of light sails. Additionally, a broadband metalens could also be used in conjunction with ultrafast solar sail systems to alleviate Doppler frequency shift [9].

## Figures and Tables

**Table 1:** The detail parameters of the different OPF schemes.

Refs	Probes		Incident Light			Medium	P_z_max_ [Pa] ^2^
	Type	Radius [μm]	Type	Wavelength [μm]	Power [mW/μm^2^] ^1^		
[12]	Polystyrene sphere	2.03	Bessel	1.064	1	Air/Water	−3.9 × 10^−3^
[19]	SiO_2_ triangular prism	5 ^3^	Multiple beams	0.532	785	Air/Water	−4.6 × 10^−2^
[20]	Si_3_N_4_ sphere	0.04	Femtosecond laser pulses	0.42	5000	CS_2_ solvent	−398
[27]	Chiral metamaterial	0.19 ^4^	RCP beam	1.55	20	Vacuum	−70
[44]	Plasmonic sphere	0.08	Bessel beam	0.39~0.48	100	Water	−50
[45]	Gain sphere	0.06	Plane wave	0.457 ^5^	1	Air	−11
This work	Metalens	10	LCP beam	1.517~2.117	0.25	Vacuum	−9 × 10^−2^

^1^ To keeping our units the same, we do mW/μm^2^. ^2^ The Maximum optical pressure along the *z*-axis. ^3^ The side length and height. ^4^ The thickness. ^5^ The pump wavelength.

**Figure A1:**
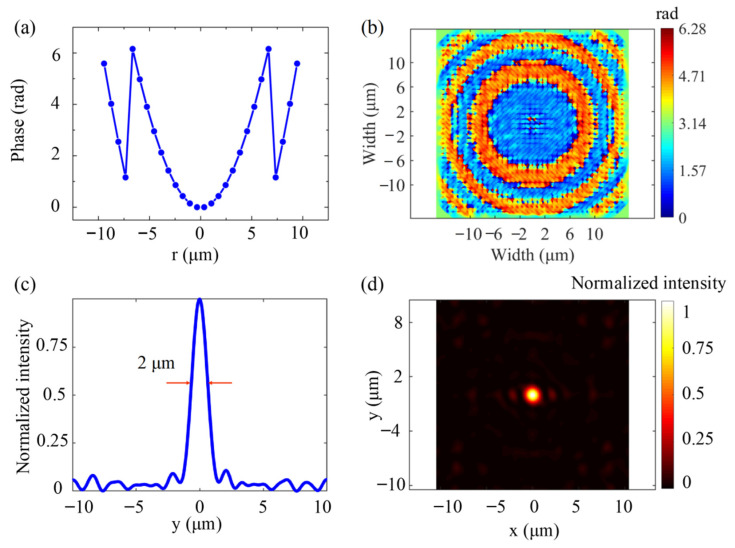
The realization of the metalens. (**a**) The hyperbolic phase distribution of metalens with sampling at the centre of each unit cell. (**b**) The output near-field phase distribution of metalens (*z* = 19.9 μm). (**c**) FWHM of foci (2 μm). (**d**) Simulated intensity distributions of the foci at *λ* = 1.817 μm.

**Figure A2:**
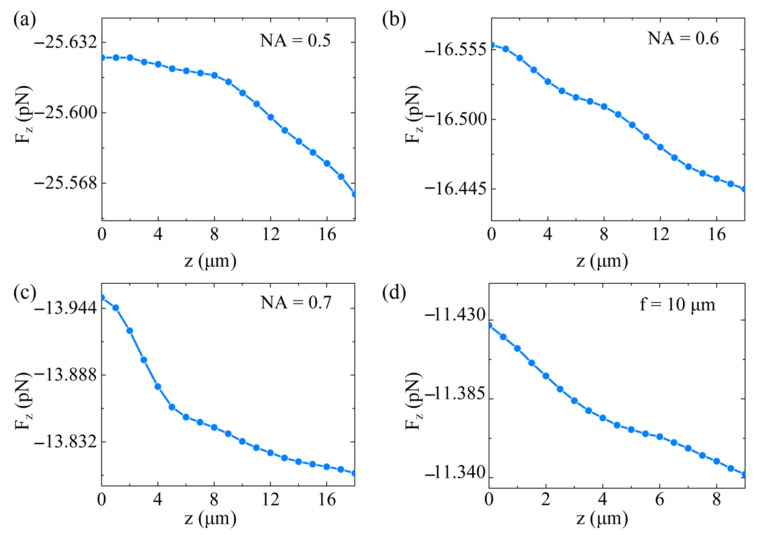
The OPF for (**a**) NA = 0.5, (**b**) NA = 0.6, (**c**) NA = 0.7, and (**d**) *f* = 10 μm, respectively. The incident light power for NA = 0.5, 0.6, and *f* = 10 μm is 1.5 W, and for NA = 0.7 is 2 W.

**Figure A3:**
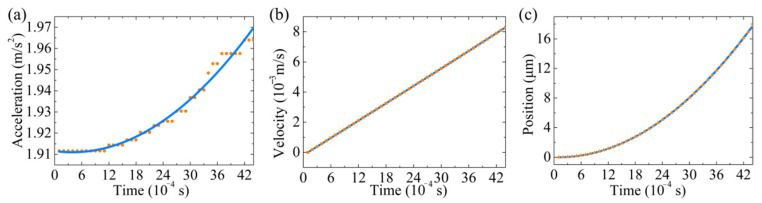
Time evolution of the equation shows the optical pulling behaviour. (**a**) The acceleration, (**b**) the velocity and (**c**) the trajectory of the metalens in the time domain.

## Data Availability

Data is contained within the article.

## Appendix A. The Theoretical Analysis of the Metalens Photonic Probe

The electromagnetic wave emitted from the metalens must constructively interfere at the focal point, similar to a conventional spherical lens. Therefore, the phase distribution of the metalens should be equal to the conventional spherical lens (see Equation (1) in the main text).

There are two ways to achieve the phase profile of the metalens, including the propagation phase and the P-B phase. The latter can get a precise phase profile via rotating the azimuthal angel *α* (*x*, *y*) of each unit cell at coordinate (*x*, *y*), which is suitable for broadband transmission. Therefore, the P-B phase is used to realize the metalens.

Let us assume that an LCP/RCP light vertically impinges on a unit cell. Its orientation direction is forming an angle *α* with the *y*-axis (see Figure 2c in main text). By applying the Jones matrix, the output light field can be expressed as [39]

(A1) $$\begin{array}{ll} {\vec{E}}_{out} & =\beta \left[\begin{array}{l} \cos^{2}\alpha \sin\alpha \cos\alpha \\ \sin\alpha \cos\alpha \sin^{2}\alpha \; \end{array}\right]{\vec{E}}_{L}\\ & =\beta \left[\begin{array}{l} \cos^{2}\alpha \sin\alpha \cos\alpha \\ \sin\alpha \cos\alpha \sin^{2}\alpha \; \end{array}\right]\left[\begin{array}{l} 1\\ \pm i \end{array}\right]\\ & =\frac{1}{2}\beta \left(\cos2\alpha +i\cdot \sin2\alpha \right)\left[\begin{array}{l} 1\\ \mp i \end{array}\right]+\frac{1}{2}\beta \left[\begin{array}{l} 1\\ \pm i \end{array}\right]\\ & =\frac{1}{2}\beta e^{i\cdot 2\alpha }{\vec{E}}_{R}+\frac{1}{2}\beta {\vec{E}}_{L} \end{array}$$

Here, ${\vec{E}}_{L/R}$ = ${\vec{e}}_{x}$ ± *i*${\vec{e}}_{y}$ only correspond to the polarization properties of the light, and the constant term 1/√2 is negligible, *β* is the propagation constant. According to Equation (A1), the output polarization including the co-polarization that only has amplitude modulation and the counter-polarization with the additional *φ* = 2*α* phase modulation. With the angle *α* rotating from 0 to π, the phase profile of the metalens can be distributed from 0 to 2π. Meanwhile, the structure parameters need to be designed to realize the high polarization conversion efficiency and *η*.

## Appendix B. The Simulation Analysis of the Metalens Photonic Probe

To well-designing the metalens, we varied the geometric parameters (*d*, *h*, *t*, *a*, *b*) of the unit cell shown in Figure 2b in the main text, seeking to find designs that could exhibit optical traction behaviour. We focus on a silicon-on-insulator (SOI) platform with Si resonators on a silica substrate that could be integrated into lightweight structures exhibiting both a high refractive index and ultra-low losses in the infrared. The current SOI processing technology is mature, conducive to subsequent fabrication and experiments. In addition, the ultrathin substrates can reduce weight effectively, which is crucial in space propulsion or manipulation applications. The high aspect ratio of the unit cell can be fabricated with Atomic Layer Deposition (ALD) method, which has been experimentally proven before [39,48]. As the designed unit cell performs well, the unit cell needs to be appropriately arranged on the substrate according to Equation (1).

We use the FEM solver COMSOL Multiphysics to simulating the metalens. Periodic boundary conditions were applied to the unit cell boundaries, and the light source is incident at the scattering boundary. In our design, A PB phase-based focusing metalens is first designed to realize the conversion that converts the collimated RCP Gaussian beam into a focusing LCP Gaussian beam. Then, a divergent LCP Gaussian light source is placed at the focal point of the metalens, and the focal radius of the light source is the same as that of the metalens. In this manner, we get the collimated RCP Gaussian beam according to the principle of reversibility of light. Once the scattering fields are obtained numerically, we can calculate the OPF by integrating the MST on a closed surface surrounding the metalens.

Based on the design process mentioned above, the designed PB phase-based focusing metalens has a width of 20 μm, the focal length *f* is 20 μm, the NA is 0.4, and the focal radius is 2 μm. The metalens is located in the positions *z* = 20 µm. The collimated RCP incident beam propagates along -*z*-direction from the substrate side with a wavelength of 1.817 μm. Figure A1a is the phase distribution versus radius *r* sampling at the centre of each unit cell. The phase distribution presents a hyperbolic, and the incident wave vectors can eventually be condensed into one point. Figure A1b shows the phase distribution in the output near-field *x*-*y* plane of the metalens (*z* = 19.9 μm). The full width at half-maximum (FWHM) of the LCP foci is 2 μm, as shown in Figure A1c. The normalized optical field intensity distribution of the LCP Gaussian beam at the focal plane is shown in Figure A1d. After that, we placed a divergent LCP Gaussian beam with a radius of 2 μm at the focal point (*z* = 0 μm). The divergent LCP Gaussian beam converses into a collimate RCP Gaussian beam when passing through the metalens, as shown in Figure 3a in the main text.

The mass of the metalens is

(A2) $$m=\pi abh\rho_{1}{\left(D/d\right)}^{2}+D^{2}t+\rho_{2}$$

where *ρ_1_* and *ρ_2_* are the mass density of the nanopillars (Si) and the substrate layer (SiO_2_), respectively. The gravity force for the metalenses with different NAs are 1.29, 2.41, 3.73, 7.22 and 12.1 pN, respectively. The gravity force is greater than the OPF under the circumstance of NA = 0.5, 0.6, 0.7 and *f* = 10 μm with the power of 100 mW in a vaccum. For these values, we increase the incident light power to 1.5 W to overcome the impact of gravity force for NA = 0.5, 0.6, and *f* = 10 μm. For NA = 0.7, the the incident light power is 2 W. The OPF are shown in Figure A2a–d.

## Appendix C. The Dynamic Equation of the Metalens Photonic Probe

To highlight the dynamics of the metalens, we now restrict our analysis to the *y**-z* plane, where the only motion considered here is the one along the -*z*-direction. The dynamic trajectory of the metalens can be modelled by Newton’s equation [49]:

(A3) $$m\frac{d^{2}z}{dt^{2}}=F_{z}(z)z-mg$$

where *z*, *m* represent the position and mass of the metalens, respectively. The numerical analysis process of the dynamic behaviour of the metalens is based on calculating its position according to Equation (A3) after the fixed time interval. The next velocity and position of the microsphere are derived from its final velocity and position over the previous time interval ∆*t*:

(A4) $$\left\{ \begin{array}{l} v(t+\Delta t)=v(t)+\overset{\cdot \cdot }{r}(t)\Delta t\\ r(t+\Delta t)=r(t)+v(t)\Delta t \end{array}\right.$$

To characterize the dynamics of the metalens under divergent LCP Gaussian beam illumination, we numerically calculated the Equations (A3) and (A4) in time. Figure A2 shows the related dynamic parameters, including acceleration, velocity and position in the time domain. The gravity force is 2.41 pN. The metalens is move in a vacuum, so we did not consider the influence of air resistance force.We assume that the metalens and the light source are initially located in the positions *z* = 20 µm and *z* = 0 µm at time *t* = 0 s. For this condition, the centre-of-mass trajectory shows that the metalens accelerate toward the light source parallel to the beam axis.
